# Influence of CYP2C19 genetic variants and smoking on dual antiplatelet efficacy in patients with coronary artery disease

**DOI:** 10.3389/fcvm.2023.1105001

**Published:** 2023-01-24

**Authors:** Yujing Cheng, Yan Sun, Dai Zhang, Xiaoteng Ma, Chi Liu, Chengping Hu, Tienan Sun, Ziwei Zhao, Xiaoli Liu, Yujie Zhou

**Affiliations:** ^1^Beijing Key Laboratory of Precision Medicine of Coronary Atherosclerotic Disease, Department of Cardiology, Beijing Anzhen Hospital, Beijing Institute of Heart Lung and Blood Vessel Disease, Clinical Center for Coronary Heart Disease, Capital Medical University, Beijing, China; ^2^Department of Cardiology, Beijing Friendship Hospital, Capital Medical University, Beijing, China

**Keywords:** coronary artery disease, antiplatelet therapy, clopidogrel, CYP2C19, smoking

## Abstract

**Introduction:**

This study aimed to investigate the effects of smoking and CYP2C19 gene polymorphism on antiplatelet therapy to specify the most optimized and accurate antiplatelet therapy for different populations.

**Methods:**

This study included 6,353 patients with coronary artery disease (CAD). In total, 2,256 (35.5%) were smokers and 4,097 (64.5%) were non-smokers. Patients carrying a CYP2C19*2 or *3 allele were considered loss-of-function (LOF) allele carriers. The medical history of patients who had undergone percutaneous coronary intervention (PCI) at Beijing Anzhen Hospital was recorded. The primary endpoint was major adverse cardiovascular or cerebrovascular events (MACCE) during the 6-month follow-up period. A Cox regression model was used to assess the interactions between antiplatelet efficacy and CYP2C19 LOF allele carrier status, stratified by smoking status.

**Results:**

Compared to clopidogrel plus aspirin, ticagrelor plus aspirin reduced the MACCE recurrence risk in non-smokers (carrier: 6.0 vs. 2.0%, hazard ratio 0.298, 95% confidence interval 0.204–0.635, *P* < 0.0001; non-carrier: 5.8 vs. 2.1%, hazard ratio 0.358, 95% confidence interval 0.189–0.678, *P* = 0.002), and not in smokers. Similar results were discovered regarding the recurrence rate for hospitalization for ischemic cardiac events in non-smokers. No apparent difference was discovered in the bleeding events in either group. There were no significant associations between antiplatelet medication and CYP2C19 LOF allele carrier status for the MACCE recurrence risk among smokers (*P* = 0.943, respectively) or non-smokers (*P* = 0.774, respectively).

**Conclusion:**

In patients with CAD after PCI, ticagrelor plus aspirin lowered the MACCE recurrence risk in CYP2C19 LOF allele carriers and non-carriers compared with clopidogrel plus aspirin alone among non-smokers. The efficacy of antiplatelet therapy varies between CYP2C19 LOF allele carrier status. No significant interaction between CYP2C19 LOF allele carrier status and antiplatelet effectiveness was observed. However, caution should be used to interpret our results considering the many limitations of our investigation.

## 1. Introduction

Dual antiplatelet therapy (DAPT), which consists of aspirin and a P2Y12 receptor inhibitor, has become the cornerstone for preventing recurrent ischemic events in patients undergoing percutaneous coronary intervention (PCI).

Despite receiving standardized clopidogrel treatment, 20–30% of patients with coronary artery disease (CAD) continue to exhibit low, or even no response, to clopidogrel, which is referred to as clopidogrel resistance. The responsiveness of platelets to clopidogrel is determined by genotype (1). Cytochrome P450 (CYP450) enzyme-related CYP2C19 contains at least 25 allelic variants, including gain-of-function and loss-of-function (LOF) alleles, and deletion variants are directly related to clopidogrel resistance. Among Asians, the most common LOF gene is CYP2C19*2 (rs4244285) (2). Mega et al. showed that a CYP2C19*2 allele mutation can increase the risk of major adverse cardiovascular or cerebrovascular events (MACCE) (3). Therefore, a warning was issued in 2010 by the United States Food and Drug Administration regarding clopidogrel. The genotype was tested to confirm patients’ clopidogrel metabolism and risk of adverse reactions and genetic polymorphisms. Three multicenter, randomized, controlled clinical trials published in the past 3 years—PHARMCLO (4), POPular-Genetics (5), and TAILOR-PCI (6)—provide important insights into the efficacy and safety of genotype-guided antiplatelet therapy strategies. According to the latest guideline, the use of prasugrel or ticagrelor in patients with CYP2C19*2 or *3 allele lowered the risk of major adverse cardiovascular events compared with conventional treatment strategies (7). Whereas, most of the patients included in these studies were Europeans and Americans, with a low percentage of Asians. No large-scale Asian cohorts have been reported before.

Gene polymorphisms are essential to the transformation and efficacy of clopidogrel. However, smoking as an independent risk factor for CAD, could enhance the drug responsiveness of clopidogrel in patients with acute coronary syndrome (ACS) (8, 9), which indicated that smokers could get greater clinical benefit compared with non-smokers, further indicating a correlation between smoking and CYP2C19 gene polymorphisms and the efficacy of clopidogrel treatment. The specific mechanism remains unclear.

This study mainly explored the correlation between CYP2C19 gene polymorphism and smoking, and the effect on antiplatelet therapy in PCI patients to identify the most optimized and accurate antiplatelet regimen for different populations.

## 2. Materials and methods

### 2.1. Study population

This was an ambispective cohort study. We identified 6,995 patients who underwent PCI at the cardiology department of Beijing Anzhen Hospital between August 2019 and March 2020, and contacted them between February 2020 and September 2020 to obtain follow-up results. Inclusion criteria were: (1) diagnosis of CAD and (2) successful PCI. Exclusion criteria included: (1) lack of baseline characteristics and/or missed follow-up data; (2) history of acute decompensated heart failure, cardiogenic shock, chronic infectious disease, or advanced cancer; (4) kidney replacement treatment or impaired kidney function with estimated glomerular filtration rate (eGFR) below 30 ml / (min × 1.73 m^2^); and (5) serious liver dysfunction, with alanine transaminase and/or aspartate transaminase ≥5 times the respective upper reference limits. Diabetes mellitus was defined as: (1) fasting blood glucose level >7.0 mmol/L, or (2) positive oral glucose tolerance test result, or (3) random blood glucose level >11.1 mmol/L with typical symptoms of diabetes. Smoking status was defined as smoking consecutively or cumulatively for >6 months in a lifetime, and cigarette smoking within 30 days before admission. The definition of dyslipidemia is elevated cholesterol, elevated low-density lipoprotein cholesterol, reduced high-density lipoprotein cholesterol, elevated triglycerides, or a combination of them. And patients with serum uric acid >420 μmol/L in men, or >357 μmol/L in women were diagnosed as hyperuricemia. All variables were obtained from the electronic medical system. This study was approved by the Institutional Review Board (approval no. 2019028), and informed consent was obtained from all participants. All patients are presented anonymously.

### 2.2. Genotyping

CYP2C19 genotyping was performed using the MassArray platform (Sequenom, San Diego, CA, USA). The genotyping success rate was >95%. Patients carrying the *2 or *3 alleles were considered LOF allele carriers. Patients without these alleles were defined as LOF allele non-carriers (10).

### 2.3. Outcomes

The primary clinical efficacy outcome was major adverse cardiovascular or cerebrovascular events (MACCE), which included all-cause death, non-fatal stroke, non-fatal myocardial infarction, and hospitalization for ischemic cardiac event. The secondary clinical safety outcome was any bleeding assessed using the Bleeding Academic Research Consortium (BARC) criteria.

### 2.4. Statistical analysis

Patients included in this study were first divided into two categories based on smoking status, and then subdivided into two subgroups based on their CYP2C19 LOF allele carrier status: carrier and non-carrier. Baseline characteristics were compared between the two subgroups in each category. Continuous variables are presented as mean ± SD. Chi-squared and Fisher’s exact tests were used to analyze categorical variables. Student’s *t*-test was used to analyze normally distributed continuous variables. Kaplan–Meier survival analyses were used to evaluate the MACCE rate throughout the 6-month follow-up period. Discrepancies between subgroups were compared using a log rank test. A Cox proportional hazards analysis was used to examine intercategory differences between the MACCE recurrence rate and bleeding events over the 6-month follow-up period; presented as a hazard ratio (HR) and 95% confidence interval (CI). Multivariable analyses adjustments were performed for age, sex, overweight, diabetes, and history of β-blocker use. A Cox proportional hazards analysis was used to assess the interaction between antiplatelet treatment and CYP2C19 LOF carrier status. A two tailed *P*-value < 0.05 showed statistical significance. SPSS (version 25.0; IBM, IL, USA) was used for analysis.

## 3. Results

### 3.1. Baseline characteristics and outcomes

Of the 6,995 patients enrolled in the study, 642 (9.2%) were lost to follow-up. Finally, 6,353 patients were selected, with a mean age of 60 ± 10 years; 2,256 (35.5%) were smokers and 4,097 (64.5%) were non-smokers. CYP2C19 LOF allele carriers comprised 55.0% of smokers (1,241 of 2,256) and 54.9% of non-smokers (2,248 of 4,097). All patients in the study were Han Chinese. Table 1 summarizes the baseline characteristics for each patient. During the 180-day follow-up period, 343 (5.4%) patients experienced at least one MACCE, and 45 (0.7%) experienced at least one major bleeding event (BARC defined bleeding events, types 2, 3, and 5) (Table 1).

**TABLE 1:** MACCE and bleeding events.

	Amount	Percentage
**Major cardiovascular and cerebrovascular ischemic events**		
All-cause death	32	0.5
Non-fatal stroke	21	0.3
Non-fatal myocardial infarction	223	3.5
Hospitalization for ischemia cardiac event	280	4.4
Total	343	5.4
**Bleeding events**		
BARC type 1	856	13.5
BARC type 2	29	0.5
BARC type 3a	3	0.04
BARC type 3b	5	0.07
BARC type 3c	4	0.06
BARC type 5a	4	0.06
BARC type 5b	0	0
Total	901	14.2

BARC, Bleeding Academic Research Consortium; MACCE, major adverse cardiovascular or cerebrovascular events.

Baseline characteristics of enrolled patients are presented in Table 2. The proportion of overweight, diabetes mellitus, and β-blockers administered was higher in CYP2C19 LOF allele carriers among smokers. No other significant differences were observed between carriers and non-carriers in each group.

**TABLE 2:** Baseline characteristics of each patient with and without CYP2C19 loss-of-function alleles stratified by smoking status.

	Smokers		*P*-value	Non-smokers		*P*-value
	Carrier (*N* = 1,241)	Non-carrier (*N* = 1,015)		Carrier (*N* = 2,248)	Non-carrier (*N* = 1,849)	
Age (years)	56.84 ± 9.69	56.65 ± 9.40	0.636	61.68 ± 9.95	61.48 ± 9.74	0.521
Male sex, *n* (%)	1,190 (95.9)	976 (96.2)	0.829	1,426 (63.4)	1,193 (64.5)	0.432
Overweight, *n* (%)	783 (63.1)	689 (67.9)	0.019	1,422 (63.3)	1,135 (61.4)	0.256
Diabetes mellitus, *n* (%)	456 (58.2)	328 (41.8)	0.029	810 (36.0)	691 (37.4)	0.361
Hypertension, *n* (%)	843 (67.9)	663 (65.3)	0.193	1,631 (72.6)	1,352 (73.1)	0.621
Hyperuricemia, *n* (%)	323 (26.0)	244 (24.0)	0.284	557 (24.8)	471 (25.5)	0.587
Dyslipidemia, *n* (%)	976 (78.6)	794 (78.2)	0.837	1,690 (75.2)	1,347 (72.9)	0.123
TC (mmol/L)	4.15 ± 1.03	4.10 ± 1.03	0.209	4.15 ± 1.02	4.14 ± 1.04	0.731
HDL-C (mmol/L)	1.03 ± 0.23	1.02 ± 0.23	0.328	1.10 ± 0.25	1.09 ± 0.25	0.318
LDL-C (mmol/L)	2.48 ± 0.86	2.44 ± 0.86	0.302	2.42 ± 0.84	2.42 ± 0.86	0.841
Triglycerides (mmol/L)	1.87 ± 1.46	1.94 ± 1.62	0.291	1.72 ± 1.24	1.73 ± 1.21	0.736
HGB (g/L)	142.28 ± 29.87	142.48 ± 28.67	0.875	140.64 ± 16.10	141.14 ± 15.96	0.313
WBC (10^9^/L)	7.56 ± 2.29	7.49 ± 1.97	0.479	6.73 ± 1.84	6.76 ± 1.87	0.580
Platelet (10^9^/L)	224.15 ± 59.28	221.59 ± 55.12	0.293	224.10 ± 62.34	223.47 ± 58.21	0.741
eGFR (ml/min/1.73 m^2^)	128.43 ± 28.85	129.21 ± 27.58	0.511	122.09 ± 44.07	122.19 ± 28.37	0.932
EF (%)	60.39 ± 8.50	60.73 ± 8.24	0.328	61.10 ± 8.26	60.77 ± 8.76	0.213
Drinking, *n* (%)	319 (25.7)	241 (23.7)	0.304	234 (10.4)	192 (10.4)	1.000
**PCI indication**						
ACS, *n* (%)	985 (79.4)	803 (79.1)	0.917	1,967 (87.5)	1,624 (87.8)	0.632
CCS, *n* (%)	256 (20.6)	212 (20.9)	0.917	244 (12)	191 (10.3)	0.632
Prior stroke, *n* (%)	54 (4.4)	59 (5.8)	0.121	122 (5.4)	98 (5.3)	0.152
Prior myocardial infarction, *n* (%)	169 (13.6)	126 (12.4)	0.415	291 (12.9)	244 (13.2)	0.816
Prior PCI, *n* (%)	276 (22.2)	229 (22.6)	0.879	560 (24.9)	498 (26.9)	0.132
ACEI/ARB, *n* (%)	557 (45.7)	444 (44.8)	0.699	993 (44.4)	839 (45.4)	0.467
β-Blocker, *n* (%)	785 (64.3)	586 (59.1)	0.014	1,403 (62.7)	1,167 (63.1)	0.672
Statin, *n* (%)	1,211 (99.3)	976 (98.5)	0.099	2,207 (98.6)	1,819 (98.4)	0.784
Aspirin, *n* (%)	1,241 (100)	1,015 (100)	–	2,248 (100)	1,849 (100)	–
Triple-vessel coronary lesions, *n* (%)	183 (14.7)	133 (13.1)	0.273	351 (15.6)	256 (13.8)	0.122
Left main lesion, *n* (%)	36 (2.9)	43 (4.2)	0.106	88 (3.9)	62 (3.4)	0.359
CTO, *n* (%)	245 (19.8)	204 (20.2)	0.832	408 (18.2)	349 (19.0)	0.517
Number of stents	1.51 ± 1.00	1.54 ± 1.02	0.560	1.50 ± 1.01	1.48 ± 1.01	0.556
Average stent diameter (mm)	2.66 ± 1.03	2.69 ± 1.04	0.553	2.60 ± 1.06	2.58 ± 1.07	0.426
Total stent length (mm)	34.40 ± 25.72	35.97 ± 26.62	0.156	34.70 ± 26.58	33.74 ± 26.27	0.246

Values are given as mean + SD, or frequency *n* (%). TC, total cholesterol; HDL-C, high-density lipoprotein-cholesterol; LDL-C, low-density lipoprotein-cholesterol; HGB, hemoglobin; WBC, white blood cell; eGFR, estimated glomerular filtration rate; EF, ejection fraction; PCI, percutaneous coronary intervention; ACS, acute coronary syndrome; CCS, chronic coronary syndrome; ACEI, angiotensin-converting enzyme inhibitors; ARB, angiotensin receptor blocker; CTO, chronic total occlusion.

**FIGURE 1:**
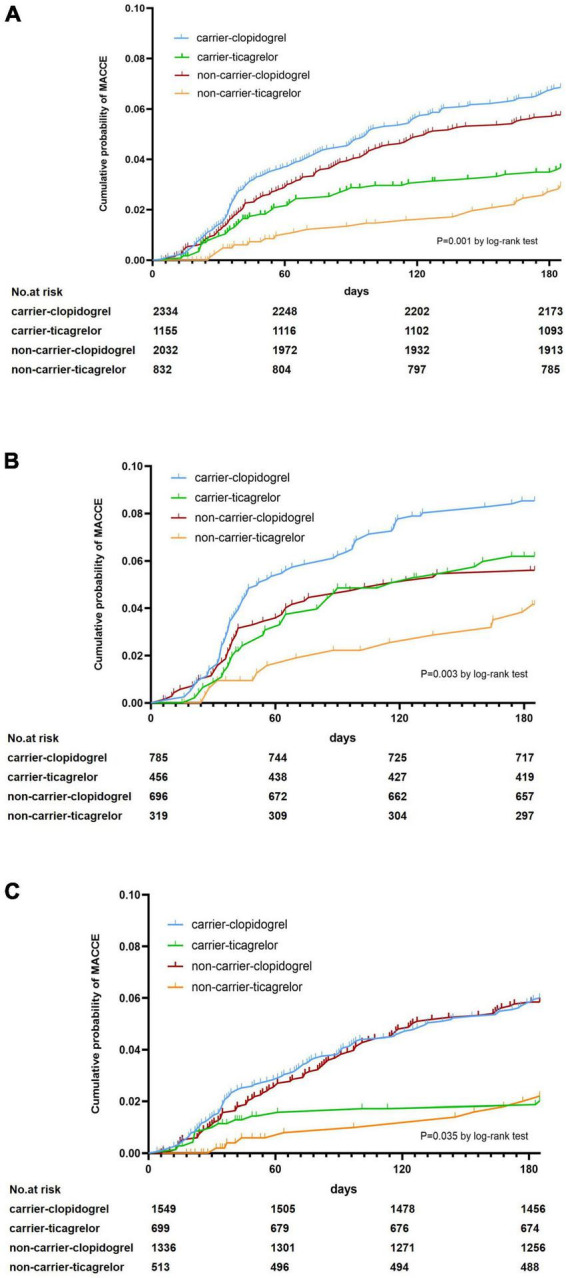
Cumulative probability of MACCE among different groups. **(A)** Cumulative probability of MACCE according to CYP2C19 loss-of-function allele carrier status and medication status. **(B)** Cumulative probability of MACCE for smokers. **(C)** Cumulative probability of MACCE for non-smokers.

Kaplan–Meier survival analyses used to calculate the cumulative probability of MACCE by carrier status and medication are presented in Figure 1A (log-rank test, *P* = 0.001). In our subgroup analysis, we found statistically significant differences in the MACCE between smokers and non-smokers (smokers log-rank test, *P* = 0.003; non-smokers log-rank test, *P* = 0.035) (Figures 1B, C).

### 3.2. Antiplatelet treatment and carrier status stratified by smoking status

As listed in Table 3, compared to clopidogrel plus aspirin, ticagrelor plus aspirin significantly reduced the MACCE recurrence risk in non-smokers (carriers: 6.0 vs. 2.0%, HR 0.332, 95% CI 0.189–0.581, *P* < 0.0001; non-carriers: 5.8 vs. 2.1%, HR 0.367, 95% CI 0.195–0.690, *P* = 0.002); however, similar effects were not seen in smokers (carriers: 8.5 vs. 6.1%, HR 0.713, 95% CI 0.459–1.109, *P* = 0.133; non-carriers: 5.6 vs. 4.1%, HR 0.815, 95% CI 0.489–1.360, *P* = 0.434). There were no significant associations between antiplatelet medication and CYP2C19 LOF allele carrier status for the MACCE recurrence risk among smokers (*P* = 0.965) or non-smokers (*P* = 0.813).

**TABLE 3:** Ticagrelor vs. clopidogrel on clinical outcomes stratified by smoking and carrier status of CYP2C19 loss-of-function alleles.

Outcomes	Smoking status	CYP2C19 loss-of-function alleles	Clopidogrel Number of patients with events/total patients (%)	Ticagrelor Number of patients with events/total patients (%)	HR (95% CI)	*P*-value	*P*-value for interaction	Adjusted HR (95% CI)	Adjusted *P*-value	Adjusted *P*-value for interaction
MACCE	Smokers	Carrier	67 (8.5)	28 (6.1)	0.713 (0.459–1.109)	0.133	0.965	0.699 (0.446–1.094)	0.117	0.943
		Non-carrier	39 (5.6)	13 (4.1)	0.815 (0.489–1.360)	0.434		0.760 (0.402–1.436)	0.398	
	Non-smokers	Carrier	93 (6.0)	14 (2.0)	0.332 (0.189–0.581)	<0.0001	0.813	0.298 (0.204–0.635)	<0.0001	0.774
		Non-carrier	78 (5.8)	11 (2.1)	0.367 (0.195–0.690)	0.002		0.358 (0.189–0.678)	0.002	
All-cause death	Smokers	Carrier	3 (0.4)	3 (0.7)	1.724 (0.348–8.542)	0.505	0.433	2.106 (0.348–12.754)	0.418	0.428
		Non-carrier	8 (1.1)	1 (0.3)	0.272 (0.034–2.174)	0.219		0.344 (0.042–2.830)	0.321	
	Non-smokers	Carrier	5 (0.3)	2 (0.3)	0.886 (0.172–4.458)	0.885	0.788	1.557 (0.274–8.852)	0.618	0.827
		Non-carrier	8 (0.6)	2 (0.4)	0.651 (0.138–3.067)	0.588		0.616 (0.127–2.987)	0.547	
Non-fatal stroke	Smokers	Carrier	2 (0.3)	5 (1.1)	4.324 (0.839–22.285)	0.08	0.906	4.752 (0.907–24.892)	0.065	0.907
		Non-carrier	1 (0.1)	0 (0)	0.028 (0.001–15,415.188)	0.693		0.001 (0.001–365.64)	0.982	
	Non-smokers	Carrier	6 (0.4)	2 (0.3)	0.739 (0.149–3.664)	0.712	0.174	0.886 (0.167–4.685)	0.886	0.166
		Non-carrier	2 (0.1)	3 (0.6)	4.041 (0.675–24.182)	0.126		4.668 (0.757–28.791)	0.097	
Non-fatal myocardial infarction	Smokers	Carrier	49 (6.2)	16 (3.5)	0.554 (0.315–0.974)	0.04	0.433	0.544 (0.307–0.963)	0.037	0.428
		Non-carrier	27 (3.9)	10 (3.1)	0.799 (0.387–1.651)	0.545		0.798 (0.383–1.659)	0.545	
	Non-smokers	Carrier	57 (3.7)	12 (1.7)	0.462 (0.248–0.862)	0.015	0.775	0.489 (0.260–0.916)	0.026	0.819
		Non-carrier	45 (3.4)	7 (1.4)	0.399 (0.180–0.884)	0.024		0.400 (0.178–0.897)	0.026	
Hospitalization for ischemia cardiac event	Smokers	Carrier	59 (7.5)	20 (4.4)	0.578 (0.348–0.961)	0.034	0.268	0.556 (0.333–0.929)	0.025	0.268
		Non-carrier	28 (4.0)	12 (3.8)	0.933 (0.475–1.835)	0.841		0.941 (0.475–1.864)	0.861	
	Non-smokers	Carrier	79 (5.1)	10 (1.4)	0.279 (0.145–0.539)	<0.001	0.379	0.294 (0.151–0.570)	<0.0001	0.399
		Non-carrier	62 (4.6)	10 (1.9)	0.163 (0.059–0.447)	<0.001		0.157 (0.057–0.434)	<0.0001	
Bleeding event^a^	Smokers	Carrier	117 (14.9)	70 (15.4)	1.131 (0.838–1.527)	0.420	0.420	1.190 (0.879–1.609)	0.260	0.465
		Non-carrier	87 (12.5)	58 (18.2)	1.372 (0.981–1.918)	0.065		1.396 (0.996–1.957)	0.053	
	Non-smokers	Carrier	226 (14.6)	91 (13.0)	0.875 (0.685–1.118)	0.286	0.869	0.854 (0.666–1.094)	0.212	0.889
		Non-carrier	188 (14.1)	64 (12.5)	0.849 (0.638–1.130)	0.263		0.847 (0.632–1.134)	0.265	
Major bleeding event^b^	Smokers	Carrier	5 (0.6)	5 (1.1)	2.158 (0.579–8.034)	0.252	0.726	2.910 (0.678–12.482)	0.151	0.676
		Non-carrier	3 (0.4)	2 (0.6)	1.452 (0.243–8.688)	0.683		1.803 (0.289–11.236)	0.528	
	Non-smokers	Carrier	13 (0.8)	5 (0.7)	0.680 (0.222–2.085)	0.500	0.862	0.821 (0.260–2.588)	0.737	0.862
		Non-carrier	6 (0.4)	6 (1.2)	1.300 (0.325–0.520)	0.710		1.271 (0.308–5.247)	0.740	

CI, confidence interval; HR, hazard ratio; MACCE, major adverse cardiovascular or cerebrovascular events.

^a^Bleeding event was defined according to the Bleeding Academic Research Consortium (BARC) criteria.

^b^Major bleeding event was defined as any bleeding event according to BARC (grades 2, 3, or 5).

The end points for non-fatal myocardial infarction and hospitalization for ischemic cardiac event showed similar outcomes. Compared to clopidogrel plus aspirin, ticagrelor plus aspirin significantly decreased the recurrence rate of non-fatal myocardial infarction and hospitalization for ischemic cardiac event in non-smokers (non-fatal myocardial infarction: carriers: 3.7 vs. 1.7%, HR 0.462, 95% CI 0.248–0.862, *P* = 0.015; non-carriers: 3.4 vs. 1.4%, HR 0.399, 95% CI 0.180–0.884, *P* = 0.024; hospitalization for ischemic cardiac event: carriers: 5.1 vs. 1.4%, HR 0.279, 95% CI 0.145–0.539, *P* < 0.001; non-carriers: 4.6 vs. 1.9%, HR 0.163, 95% CI 0.059–0.447, *P* < 0.001) and carriers in smokers (non-fatal myocardial infarction: 6.2 vs. 3.5%, HR 0.554, 95% CI 0.315–0.974, *P* = 0.04; hospitalization for ischemic cardiac event: 7.5 vs. 4.4%, HR 0.578, 95% CI 0.348–0.961, *P* = 0.034). However, smokers who were not carriers did not experience similar consequences (non-fatal myocardial infarction: 3.9 vs. 3.1%, HR 0.799, 95% CI 0.387–1.651, *P* = 0.545; hospitalization for ischemic cardiac event: 4.0 vs. 3.8%, HR 0.933, 95% CI 0.475–1.835, *P* = 0.841). There was no significant interaction between the status of CYP2C19 LOF carriers and antiplatelet medication regarding the risk of non-fatal myocardial infarction and hospitalization for ischemic cardiac event.

After adjusting for sex, overweight, diabetes mellitus, and β-blocker use, the relationships between antiplatelet medication and carrier status on the MACCE recurrence risk in non-smokers were considerably lowered, but remained no significant difference (*P* = 0.774, respectively). Multivariable adjusted models showed that ticagrelor had a more significant effect than clopidogrel in decreasing the MACCE recurrence risk (adjusted HR 0.298, 95% CI 0.204–0.635, *P* < 0.001) in non-smoker carriers.

The risk of bleeding was not increased with ticagrelor and aspirin. No interactions were observed between antiplatelet medication and carrier status on bleeding events and major bleeding events in smokers and non-smokers (smokers: bleeding event: *P* = 0.465, major bleeding event: *P* = 0.676; non-smokers: bleeding event: *P* = 0.889, major bleeding event: *P* = 0.862).

## 4. Discussion

According to the results of our study, in non-smokers, ticagrelor–aspirin antiplatelet therapy decreased the MACCE recurrence risk by 70% in carriers and 64% in non-carriers compared with clopidogrel-aspirin, without any additional risk of bleeding event. No interactions between the ticagrelor efficacy of MACCE and carrier status of the CYP2C19 LOF allele was observed in smokers or non-smokers. The mechanism underlying may be related to smoking and the CYP2C19 LOF allele affecting the efficacy of clopidogrel, without any apparent relationship with ticagrelor, as will be discussed below.

According to previous studies (11, 12), carriers of LOF alleles treated with clopidogrel had an increased incidence of major adverse cardiovascular events. Other risk factors, such as smoking, also played a role in the outcome. Some studies have revealed that smoking could enhance the efficacy of clopidogrel and improve prognosis in patients with stroke or ACS (13–16). Other studies have shown that cigarettes may lower platelet activity during treatment, and weaken the outcomes significance of CYP2C19*2 in patients with ACS (16, 17). Our study extended previous findings. We found that the difference in efficacy between clopidogrel and ticagrelor was reduced in smokers. In non-smokers, the conversion from clopidogrel to ticagrelor reduced the MACCE recurrence rate. These findings may provide inspiration for further studies on the synergistic effect between smoking and CYP2C19 gene polymorphism and clopidogrel efficacy.

The underlying mechanism between smoking and the effect on the pharmacokinetics of clopidogrel remains unclear. Previous studies have showed that smoking enhances the activity of CYP enzymes, particularly CYP2B6 and CYP1A2, thereby contributing to the high metabolism of clopidogrel (18, 19). CYP1A2 mutations have been reported to increase metabolic activity in smokers. Homozygous AA mutations have higher metabolic activity than other genotypes in smokers. In non-smokers, there was no obvious correlation between the CYP1A2 genotype and metabolism (20). According to another study, smoking increased the number of P2Y12 receptors on platelets, which enhanced the inhibitory activity of clopidogrel on platelets (21).

A study on therapies for *Helicobacter pylori* infection and CYP2C19 polymorphism revealed that smoking affected the metabolism of drugs used in eradication therapy by modifying CYP activity (22). CYP2C19 is one of the most crucial hepatic enzymes involved in clopidogrel metabolism. Smoking may have an impact on this activity; however, this unclear. Our study found that non-smokers benefited more from ticagrelor therapy than clopidogrel therapy. MACCE in the smoking group was significantly higher than in the non-smoking group, which indicated that smoking, as a traditional cardiovascular risk factor, increased the incidence of MACCE (23–27). The increased benefits of smoking on clopidogrel are unable to compensate for the damage caused by long-term and continuous smoking. For non-carriers receiving clopidogrel, the MACCE recurrence rate was lower in smokers than in non-smokers. These results indicate that smoking may influence CYP2C19 and enhance the efficacy of clopidogrel. Further investigations are required to identify the underlying mechanism, and to verify our results.

Our findings showed that ticagrelor plus aspirin was a superior antiplatelet therapy option to clopidogrel plus aspirin for non-smokers with CAD who underwent PCI. The efficacy of antiplatelet therapy varies between CYP2C19 LOF carrier status; carriers benefit more from ticagrelor. The total MACCE rate was significantly higher in smokers than in non-smokers, and there was no significant benefit of ticagrelor over clopidogrel. Therefore, smoking cessation may be the best option for lowering the MACCE recurrence rate. In future, further studies and clinical experience should be considered for individualized DAPT.

## 5. Limitation

This study had limitations. First, this research is an ambispective investigation. Compared with prospective investigation, part of the data accumulation of our investigation was not controlled by researchers, the integrity and authenticity of records directly affected the reliability of results, which would cause some biases. Second, there was a lack of quantity and years of smoking for the patients. Thus, a quantitative assessment could not be performed, and the results may have been over- or under-estimated. Third, due to technical limitations, platelet reactivity of the patients could not be directly tested at their bedside, and the components of the drug in plasma after therapy could not be directly detected. Further mechanistic and pharmacodynamic studies are required to validate our results. Finally, racial differences may have impacted the results. Different races have different risks of bleeding and ischemia. All patients in the study were Han Chinese. This result should be further verified in other regions and races.

## 6. Conclusion

In patients with CAD after PCI, ticagrelor plus aspirin could lower the recurrence risk of MACCE in both CYP2C19 LOF allele carrier and non-carrier compared with clopidogrel plus aspirin alone among non-smokers. The efficacy of antiplatelet therapy varied between CYP2C19 LOF carrier status, and carriers benefited more from ticagrelor. For smokers, smoking cessation may be the best option for lowering the MACCE recurrence rate, and ticagrelor use should be carefully considered over clopidogrel. No significant interaction between CYP2C19 LOF allele carrier status and antiplatelet effectiveness was observed.

## Data availability statement

The original contributions presented in this study are included in this article, further inquiries can be directed to the corresponding authors.

## Ethics statement

The studies involving human participants were reviewed and approved by the Institutional Review Board of Beijing Anzhen Hospital. The patients/participants provided their written informed consent to participate in this study.

## Author contributions

All authors contributed to acquisition of data, or substantial analysis and interpretation of data, and final approval of the version to be published. YZ and XL contributed to drafting the article or revising it critically for important intellectual content.
